# WHAT MATTERS MOST? REACTIONS TO THE RAISE ADVISORY COUNCIL’S NATIONAL STRATEGY TO SUPPORT FAMILY CAREGIVERS

**DOI:** 10.1093/geroni/igad104.0179

**Published:** 2023-12-21

**Authors:** Pamela Nadash, Eileen Tell, Shan Qu, Maryssa Pallis

**Affiliations:** University of Massachusetts Boston, Boston, Massachusetts, United States; ET Consulting, Belmont, Massachusetts, United States; University of Massachusetts Boston, Boston, Massachusetts, United States; University of Massachusetts Boston, Boston, Massachusetts, United States

## Abstract

This presentation reports on feedback from advocates and researchers, those working in organizations serving family caregivers, and family caregivers themselves regarding the 2022 National Strategy to Support Family Caregivers. The Strategy was issued by the RAISE Family Caregiving Advisory Council, created under the Recognize, Assist, Include, Support, and Engage (RAISE) Family Caregivers Act (2018). A Request for Information (RFI) published in the Federal Register garnered nearly 600 responses from a wide range of individuals and organizations, providing feedback about top priorities for action, topics or populations that were not adequately addressed, and recommendations for actions that could help meet stated objectives; these comments will prove critical in implementing the National Strategy, as well as in updating and revising it. This presentation sets the progress of the National Strategy in context, reports on the RFI analysis, and describes next steps, highlighting key areas of focus for the federal government, states, and other entities. Priorities included expanding the direct care workforce and increasing access to caregiver training and supports, particularly respite. Other top comments pertained to financial and workplace security for family caregivers - encouraging caregiver-friendly workplaces, paying family members to provide care, and strengthening the Family and Medical Leave Act. Respondents broadly endorsed the Strategy and noted the need for campaigns promoting greater awareness of family caregiving. The findings suggest that there is strong consensus among caregiver advocates regarding the most critical areas for action and the most favored strategies to achieve caregivers’ priority goals.

